# Supplementary material for: Engineered immunological niches to monitor disease activity and treatment efficacy in relapsing multiple sclerosis
Source: Nat Commun. 2020 Aug 3;11:3871. doi: 10.1038/s41467-020-17629-z (PMC7398910; doi:10.1038/s41467-020-17629-z)
Supplement: Supplementary file 1 — Supplementary Information [file 41467_2020_17629_MOESM1_ESM.pdf]

Engineered immunological niches to monitor disease activity and treatment efficacy in relapsing multiple sclerosis

Morris, et al.

Supplemental Figures

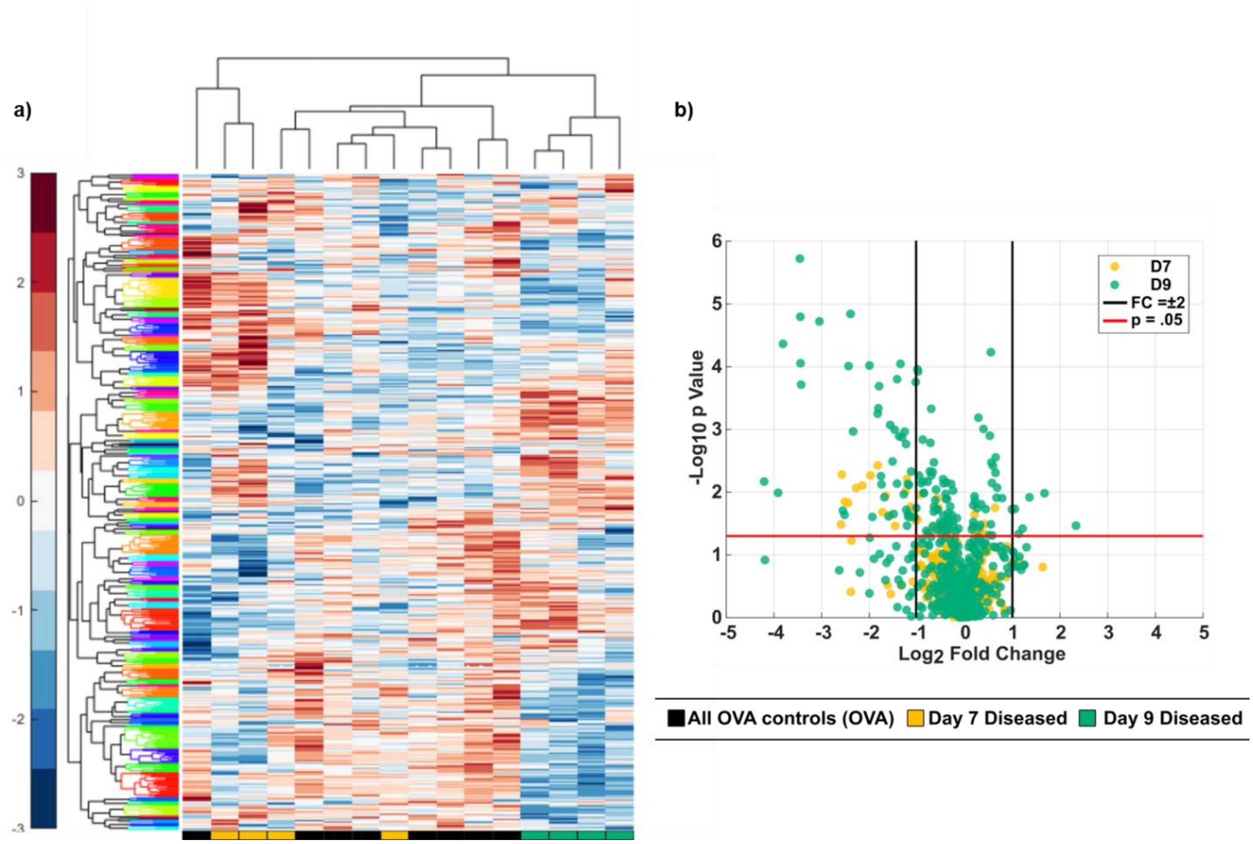

Supplementary Figure 1 Gene expression from entire OpenArray Panel. a) Heatmap and hierarchical clustering of gene expression from entire OpenArray panel (n=8 per condition, 4 at day 7, 4 at day 9). b) Volcano plot of OpenArray data. Black lines indicate a fold change of ±2 and red line indicates p = 0.05. In total, 130 genes are significantly differentially expressed at the INs between healthy and diseased mice. Statistical analysis performed by two-tailed student's t-test at individual time points.

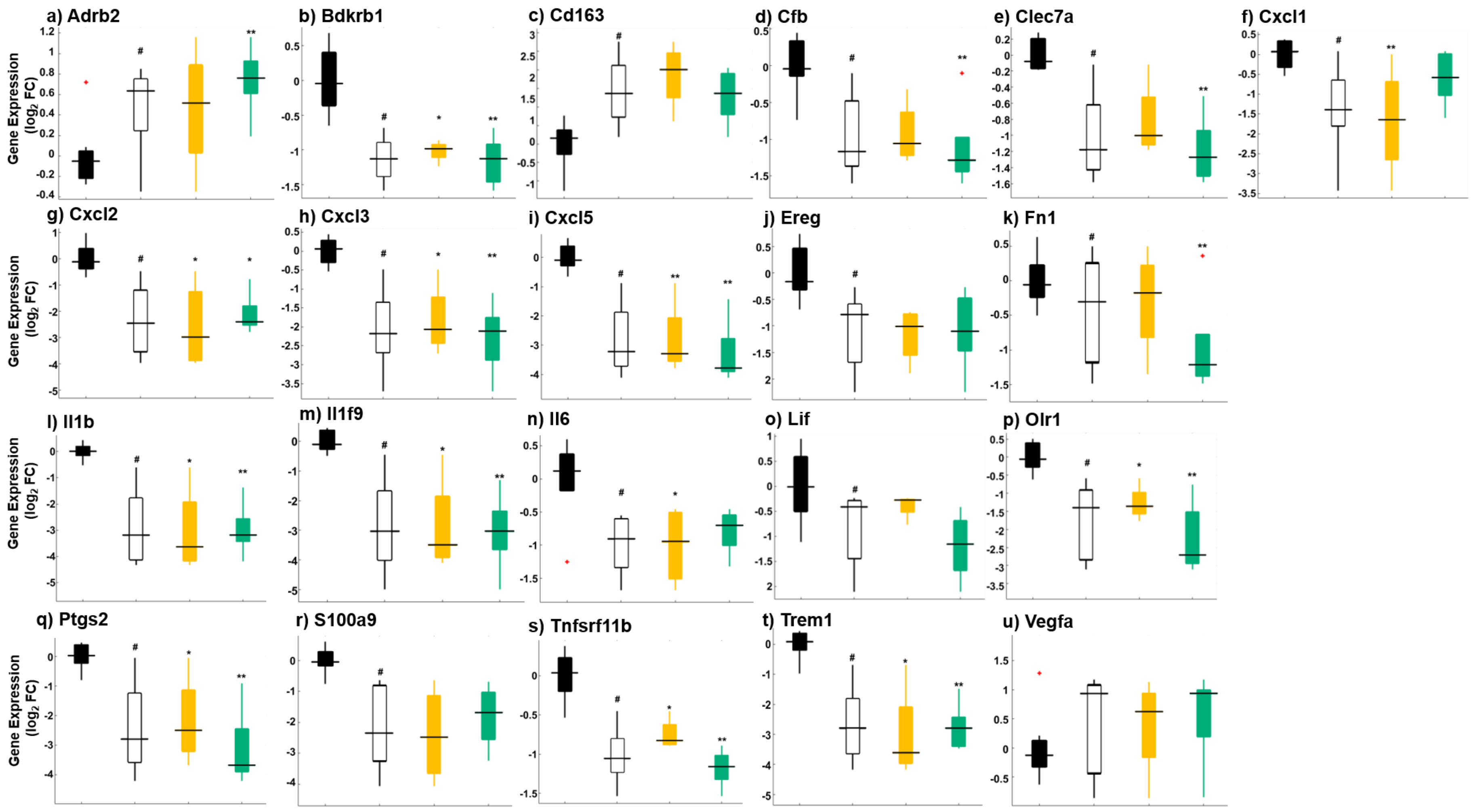

Supplementary Figure 2 Gene expression from OpenArray for 21 genes in gene signature. Log_2 FC is displayed in boxplots with 25-75th percentiles and range of data for each group displayed (outliers indicated with red +). Black and white boxes demonstrate pooled OVA controls and diseased mice, while yellow and green boxes represent the log_2 (fold change) at days 7 and 9, respectively. Two-way ANOVA was used to compare pooled OVA controls vs diseased mice (# $p<0.05$). A Bonferroni-corrected post-hoc multiple comparisons test was used to compare diseased to time matched controls (*$p<0.05$, **$p<0.005$, exact p-values in Supplementary Table 2). n=8 per condition, 4 at day 7, 4 at day 9. Box plots show the median, 25th-75th percentiles and most extreme data points not considered outliers (outliers are indicated by red +).

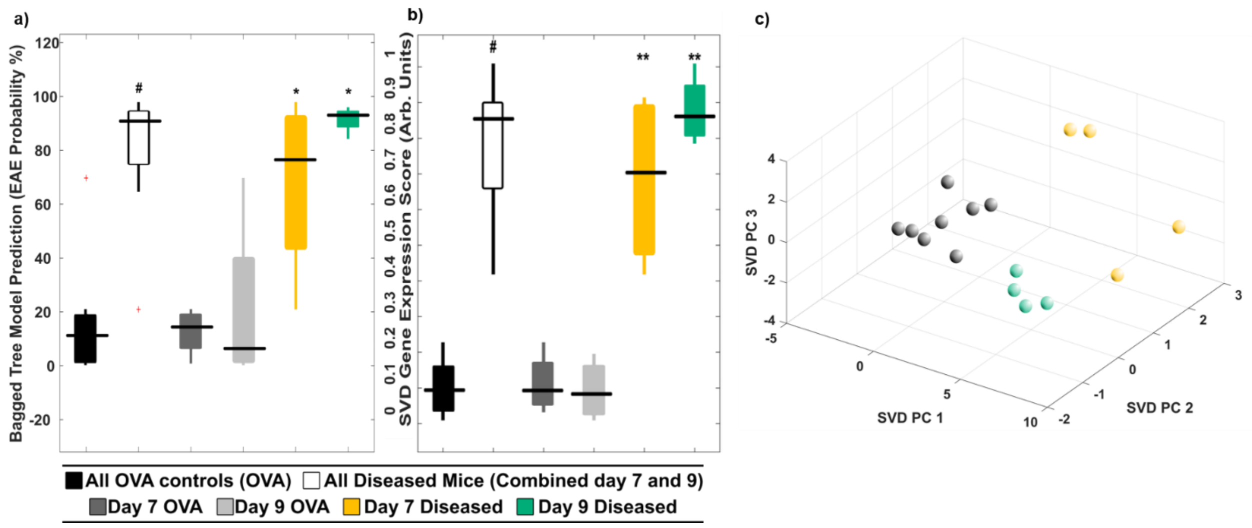

Supplementary Figure 3 Development of SVD and BT model enable classification of mice as healthy or diseased. a) Gene expression data was used to train a bootstrap aggregated (bagged) decision tree ensemble to predict the probability that any given mouse was diseased ($F(1,12) = 26.84$, #p=0.00020) b) An unsupervised dimensionality reduction technique (singular value decomposition – SVD) was used to reduce the gene expression of the 21-gene signature to a score ($F(1,12) = 85.69$, # p = 8.2e-07). Two-way ANOVA was used to compare pooled OVA controls vs diseased mice. A Bonferroni-corrected post-hoc multiple comparisons test was used to compare diseased to time matched controls (*p=0.045 at d7 and *p = 0.0086 at d9; **p = 0.00079 at d7 and *p = 4.0e-05 at d9). c) Three principle components of SVD plotted in a 3D scatter plot. The single SVD scores were computed by calculating the Euclidean distance from the OVA control centroid to any given point (each point represents an independent mouse). n=8 per condition, 4 at day 7, 4 at day 9. Box plots show the median, 25th-75th percentiles and most extreme data points not considered outliers (outliers are indicated by red +).

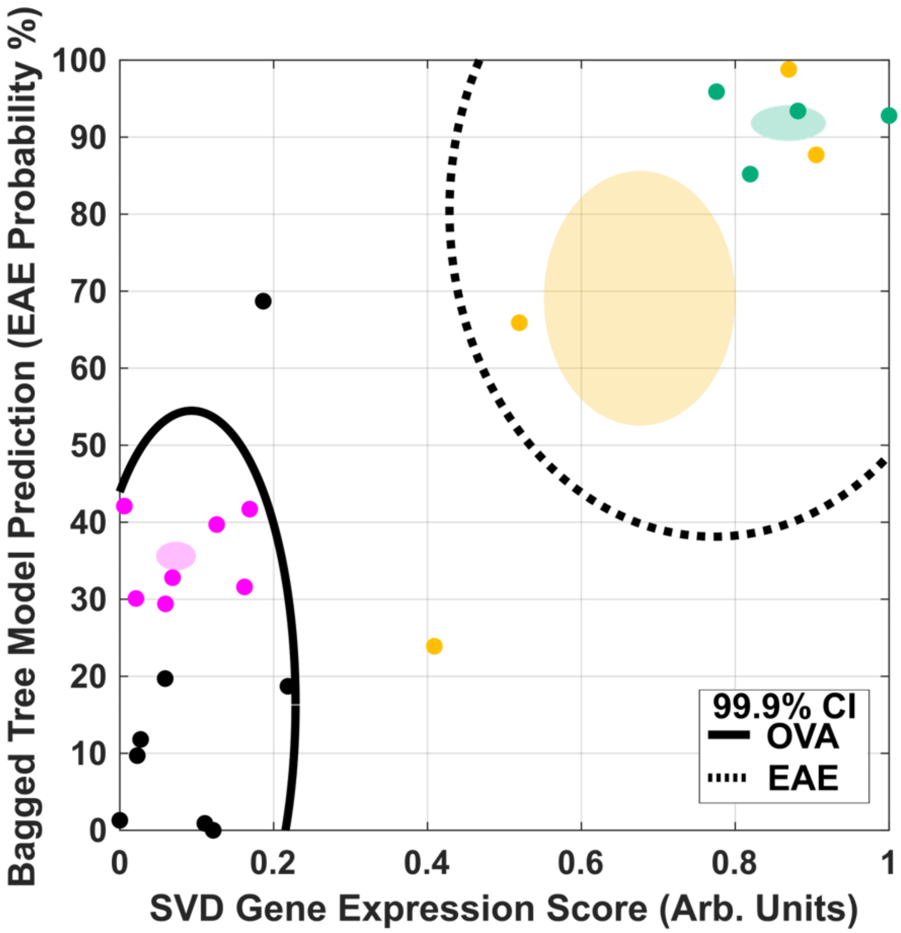

Supplementary Figure 4 INs isolated from healthy mice have similar scores to OVA controls. Plot of the bagged tree (BT) prediction score versus SVD in which pink points represent INs isolated from healthy mice (no adoptively transferred cells), black represent mice that received OVA adoptive transfers and yellow and green PLP adoptive transfers. Black lines indicate 99.9% confidence intervals for pooled diseased or control mice. Filled ovals indicate mean (centroid) and standard error of the mean for each indicated group. Clearly, the signature scores for healthy mice and OVA controls are similar.

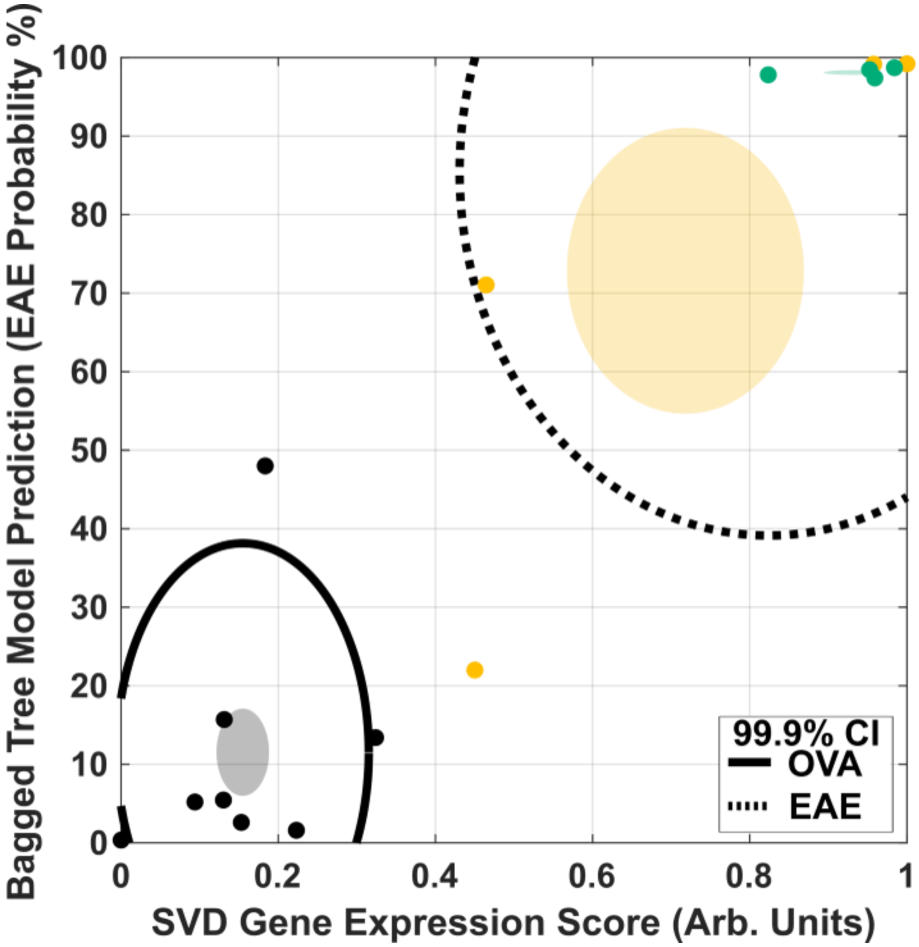

Supplementary Figure 5 Gene expression analysis by qPCR confirms OpenArray findings. The same samples examined via OpenArray were analyzed in-house by qPCR in 384 well plates and signature scores plotted as bagged tree (BT) versus SVD. Black lines indicate 99.9% confidence intervals for pooled diseased or control mice. Filled ovals indicate mean (centroid) and standard error of the mean for each indicated group. Clearly, the results via qPCR indicate similar findings to OpenArray.

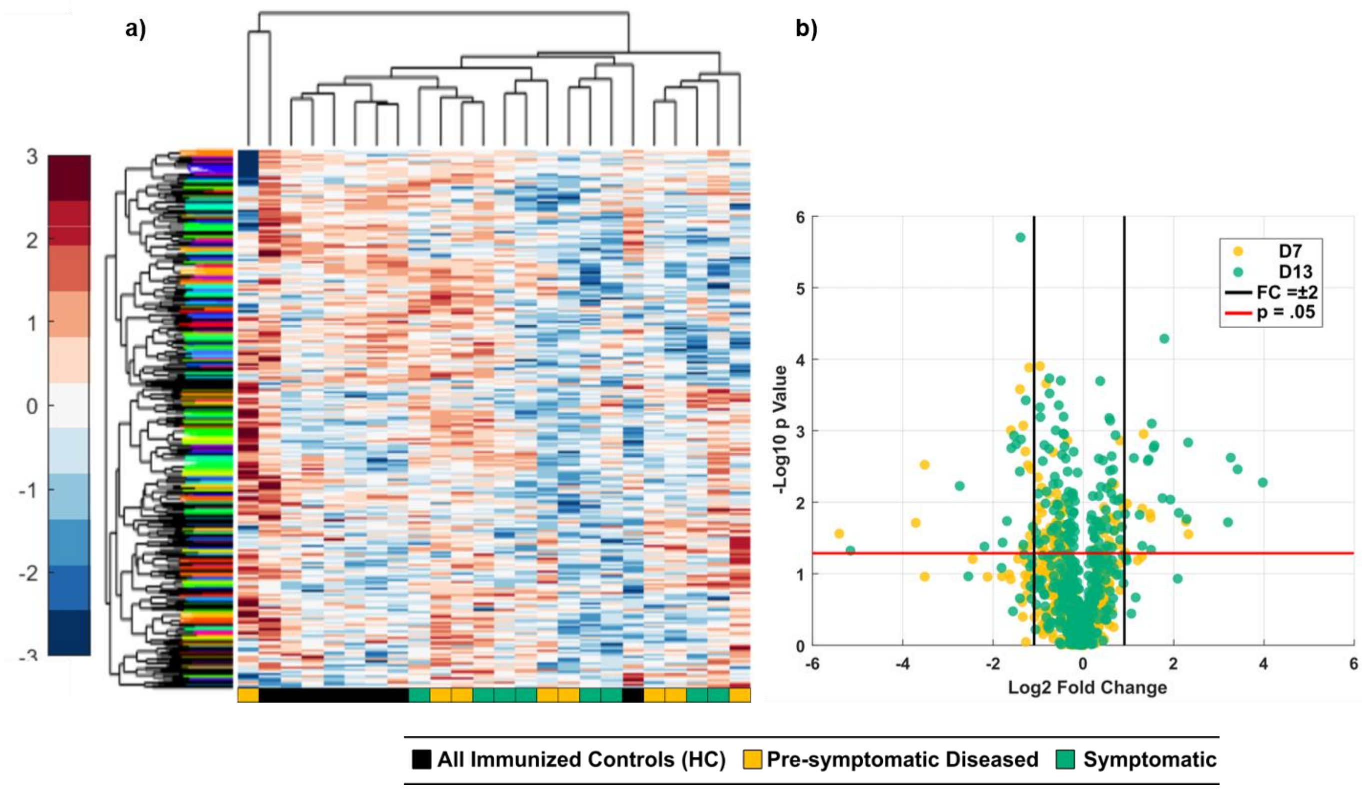

Supplementary Figure 6 Gene expression from entire OpenArray Panel for immunized mice. a) Heatmap and hierarchical clustering of gene expression from entire OpenArray panel (n=8 immunized control [4 at day 7, 4 at day 13] and n= 8 diseased [at each time point]). b) Volcano plot of OpenArray data. Black lines indicate a fold change of ±2 and red line indicates p = 0.05. In total, 222 genes are significantly differentially expressed at the INs between healthy and diseased mice. Statistical analysis performed by two-tailed student's t-test at individual time points.

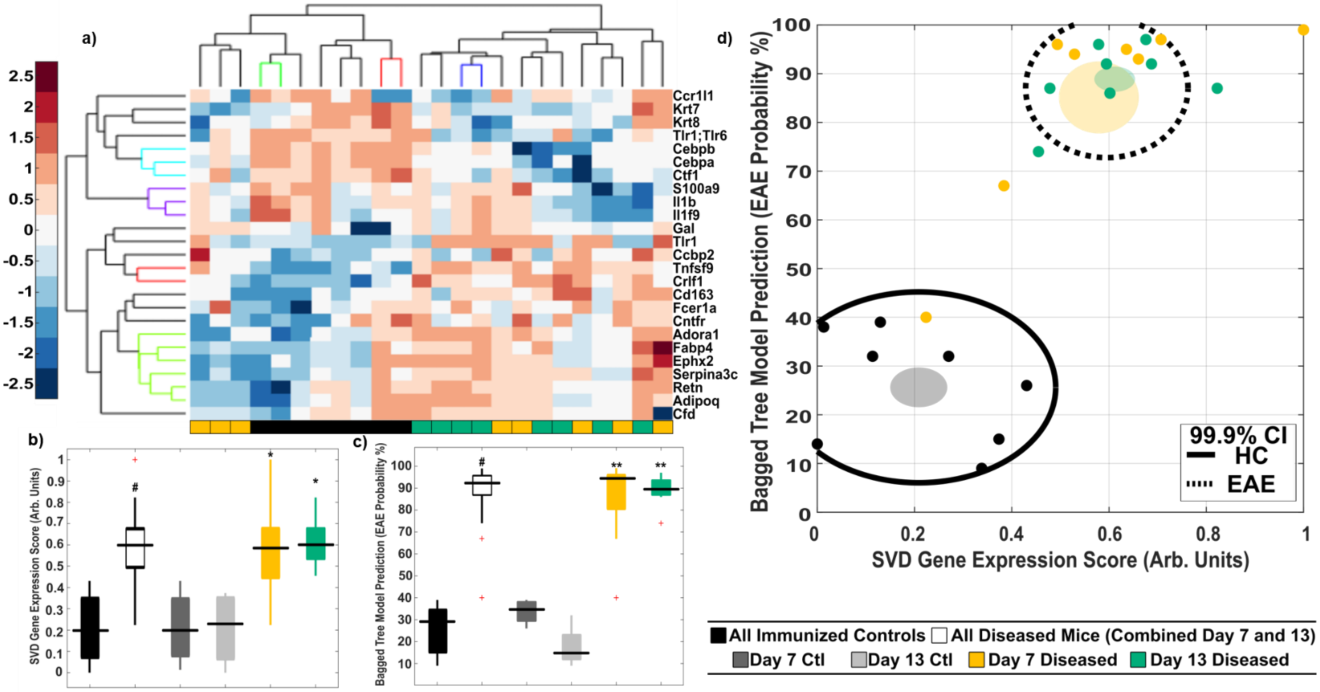

Supplementary Figure 7 IN gene expression changes enables scoring and segmenting of diseased and healthy in an immunization model of EAE. a) Heatmap and hierarchical clustering of 25 identified genes of interest, expression levels standardized by gene. b) An unsupervised dimensionality reduction technique (singular value decomposition – SVD) was used to reduce the gene expression of the 25-gene signature to a score ($F(1,20) = 23.84$, # $p = 0.000090$, *$p = 0.022$ for day 7, *$p=0.010$ for day 13). c) Gene expression data was used to train a bootstrap aggregated (bagged) decision tree ensemble to predict the probability that any given mouse was diseased ($F(1,20) = 104.70$, #$p=2.1e-9$, **$p = 3.8e-5$ for day 7, **$p = 3.2e-7$ for day 13).). Two-way ANOVA was used to compare pooled OVA controls vs diseased mice. A Bonferroni-corrected post-hoc multiple comparisons test was used to compare diseased to time matched controls. d) Plot of the bagged tree (BT) prediction score versus SVD indicates separation between groups. Black lines indicate 99.9% confidence intervals for pooled diseased or control mice. Filled ovals indicate mean (centroid) and standard error of the mean for each indicated group. Box plots show the median, 25th-75th percentiles and most extreme data points not considered outliers (outliers are indicated by red +). n=8 immunized control (4 at day 7, 4 at day 13) and n= 8 diseased (at each time point).

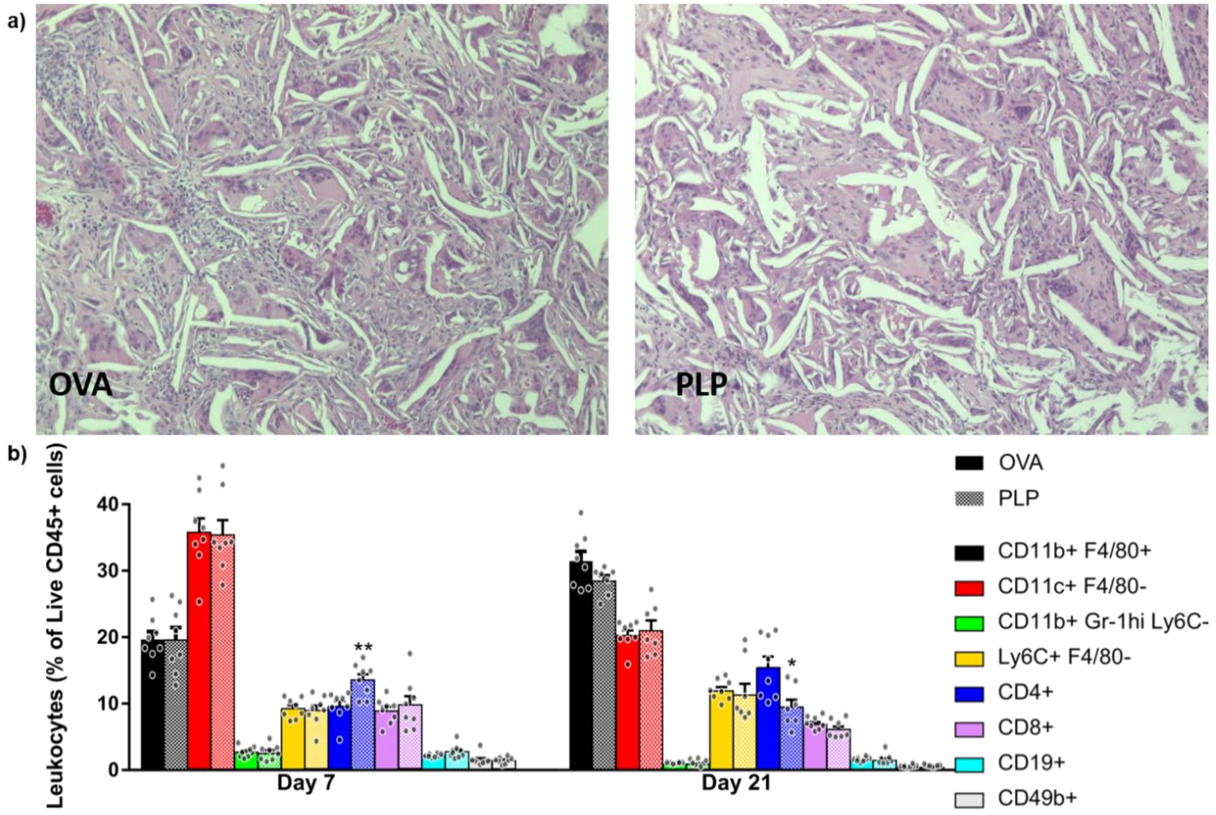

Supplementary Figure 8 Evaluation of immune cells within INs. a) H&E stains indicate a normal FBR for a porous material in both OVA and PLP conditions demonstrating immune infiltrate, foreign body giant cell (FBGC) formation, and tissue ingrowth. b) Flow cytometry for immune cell surface markers demonstrates minor differences between OVA and PLP, but little change in the cell populations within the INs over time. Data are presented as mean + SEM. Statistical analysis performed by two-tailed student's t-test at individual time points, asterisks indicate change from control (**p=0.0052, *p=0.014)). n=8 mice (day 7, day 21 OVA) and n=7 mice (day 21 PLP).

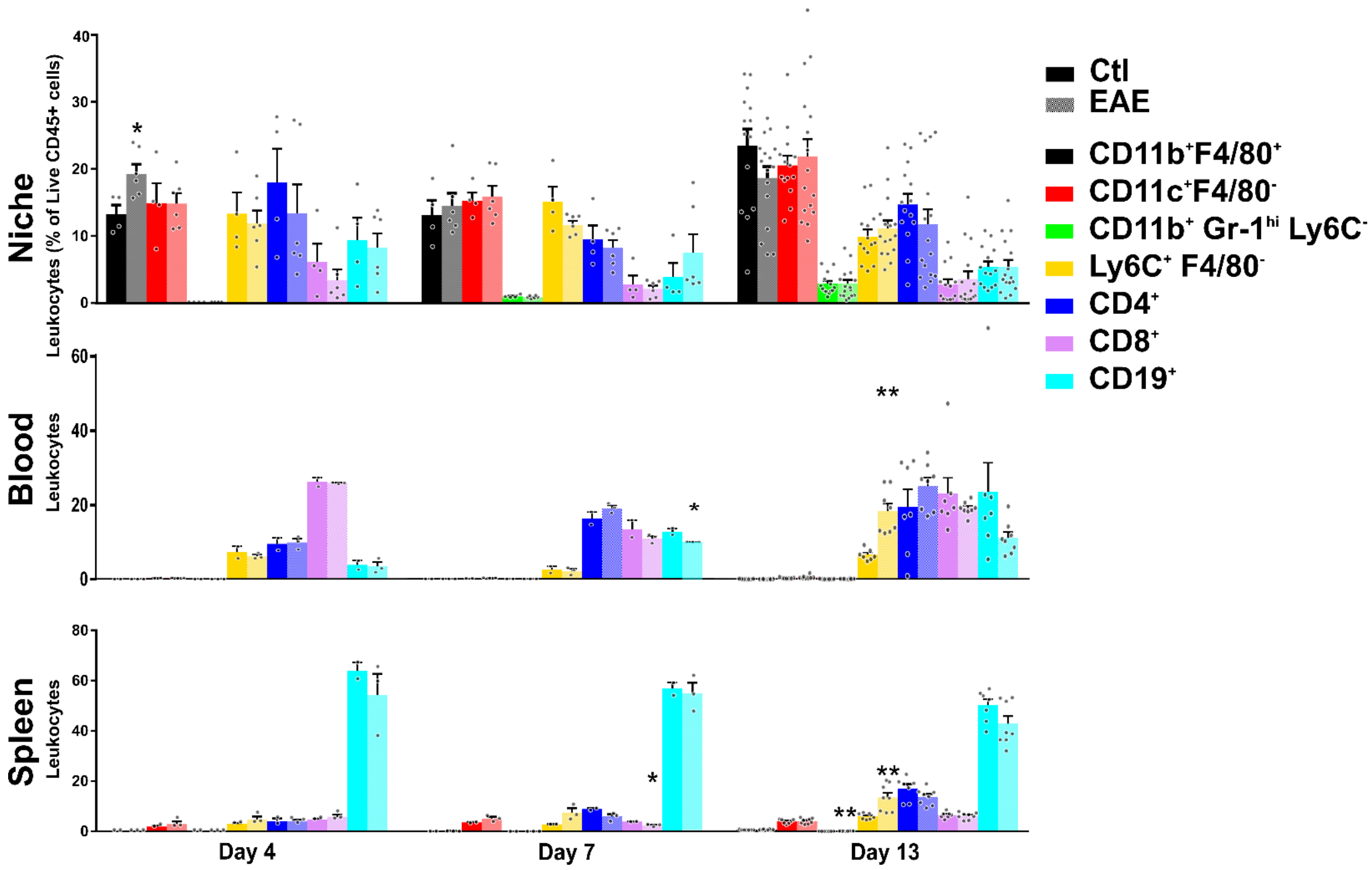

Supplementary Figure 9 Evaluation of immune cell populations during an immunization model of EAE. Flow cytometry for immune cell surface markers demonstrates cell populations through time in IN, blood, and spleens. Data are presented as mean + SEM. Statistical analysis performed by two-tailed student's t-test at individual time points, asterisks indicate change from control. (IN, day 4 *p = 0.021; blood, day 7 *p = 0.025; blood day 13 *p=0.00020; spleen day 7 *p=0.025; spleen day 13 **p=0.0023 [CD11b^{+}Gr-1^{hi}Ly6C^{-}] and 0.0034 [Ly6C^{+} F4/80^{-}]). INs at day 4 and 7: n=4 (Ctl), n=6 (EAE); at day 13: n=14 (Ctl), n=16 (EAE). Blood and spleens at day 4 and 7: n=2 (Ctl), n=3 (EAE); at day 13: n=7 (Ctl), n=8 (EAE).

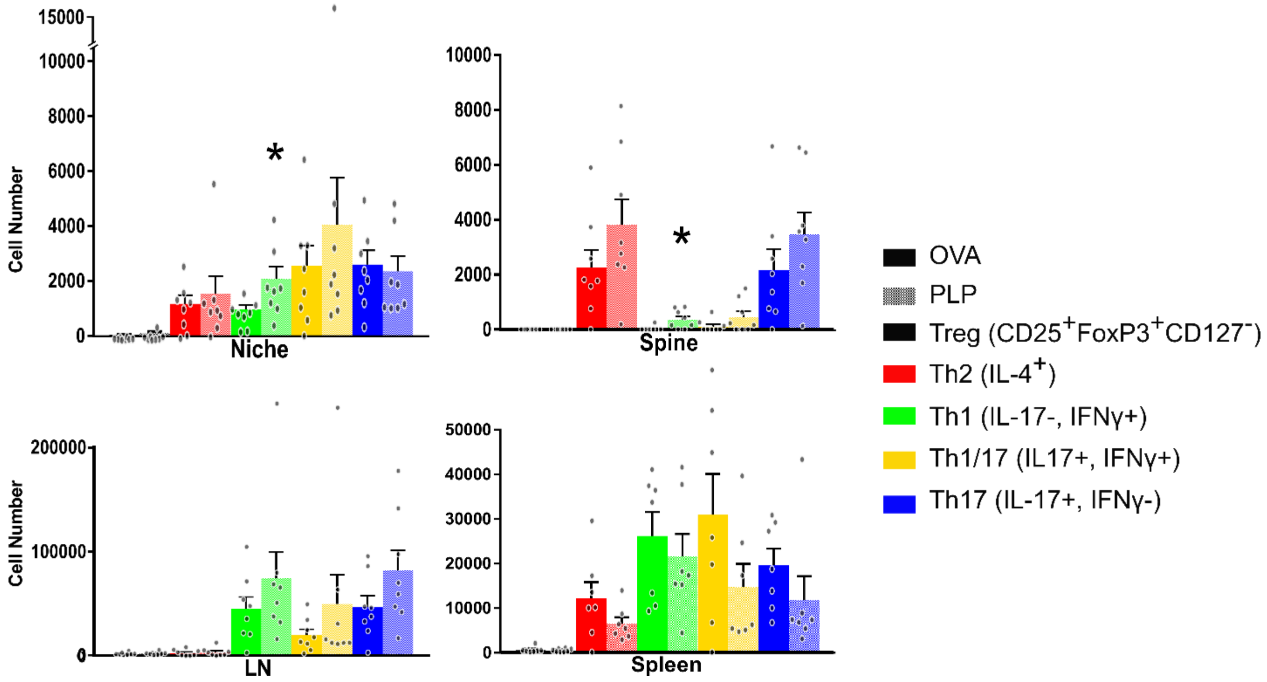

Supplementary Figure 10 Evaluation of T-cell subsets in adoptive transfer model of EAE. Flow cytometry at day 9 post-adoptive transfer demonstrates T-cell subsets present in IN, spinal cord, lymph nodes, and spleens. Data are presented as mean + SEM. Only Th1 (Il-17$^-$, IFN$^+$) cells in scaffolds and spinal cords were significantly increased at this time point. Statistical analysis performed by two-tailed student's t-test, asterisks indicate change from control for that organ (*p=0.034 for IN and *p=0.016 for Spine), (n=8).

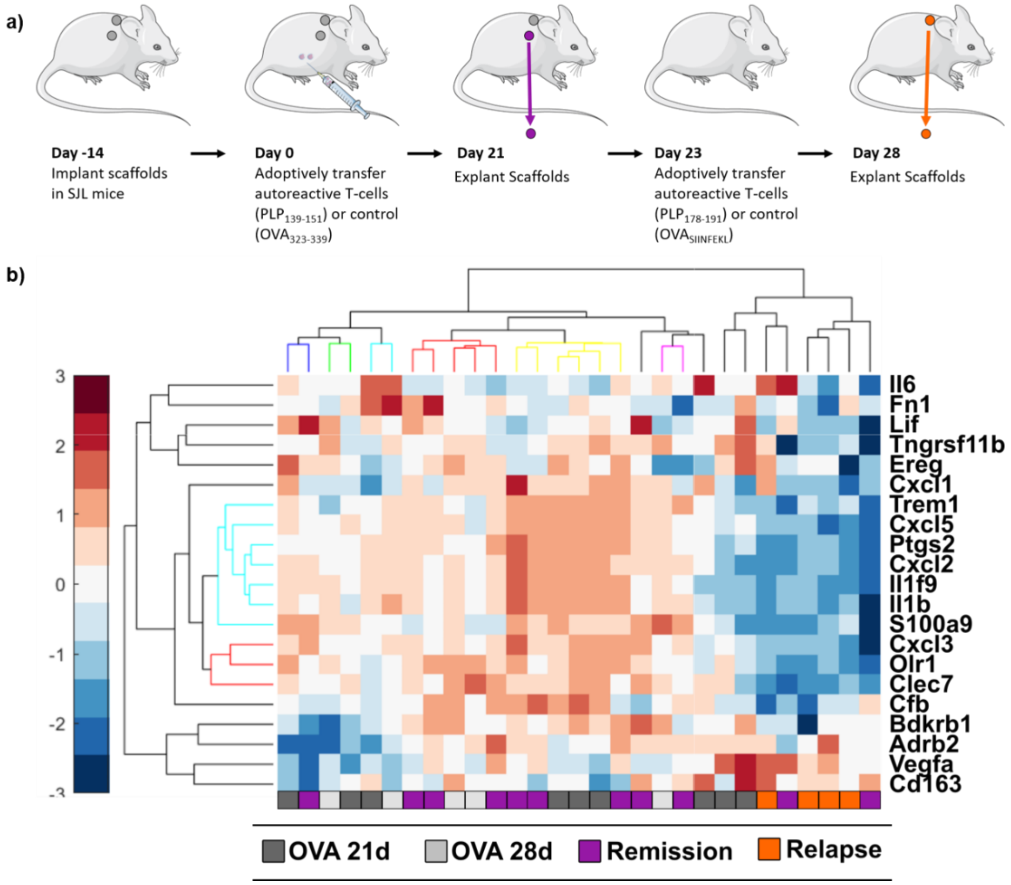

Supplementary Figure 11 Dynamics of EAE disease state are detectable in IN. a) SJL mice were implanted with PCL INs 14 days prior to adoptive transfer of T cells reactive to either $PLP_{139-151}$ (EAE) or $OVA_{323-339}$ (control). INs were subsequently removed at a remission (21 day - purple) or relapse (28 day - orange) time point for analysis via qPCR. A second adoptive transfer of cells reactive against another immunodominant epitope (to mimic epitope spreading in relapse) was performed on day 23 to induce relapse. b) Heatmap and hierarchical clustering of 21 identified genes of interest, expression levels standardized by gene. Mouse and syringe cartoon from Servier Medical Art, https://smart.servier.com/smart_image/.

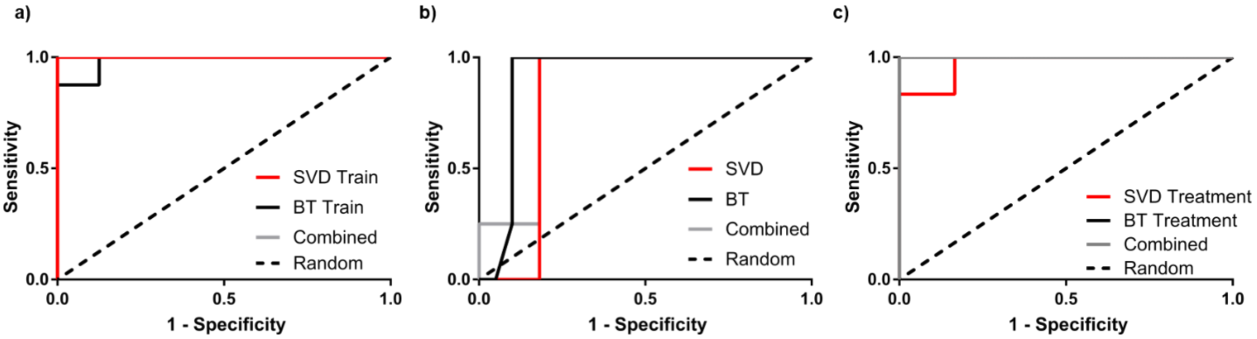

Supplementary Figure 12 ROC curves validate the signature scores from the IN as an effective diagnostic, prognostic, and treatment monitor. ROC curves were plotted for the disease onset data used

to train the signature classification model (a), relapse and remission data (b), and particle treatments (c). In each case the area under each curve is > 0.8 indicating successful diagnostic efficacy.

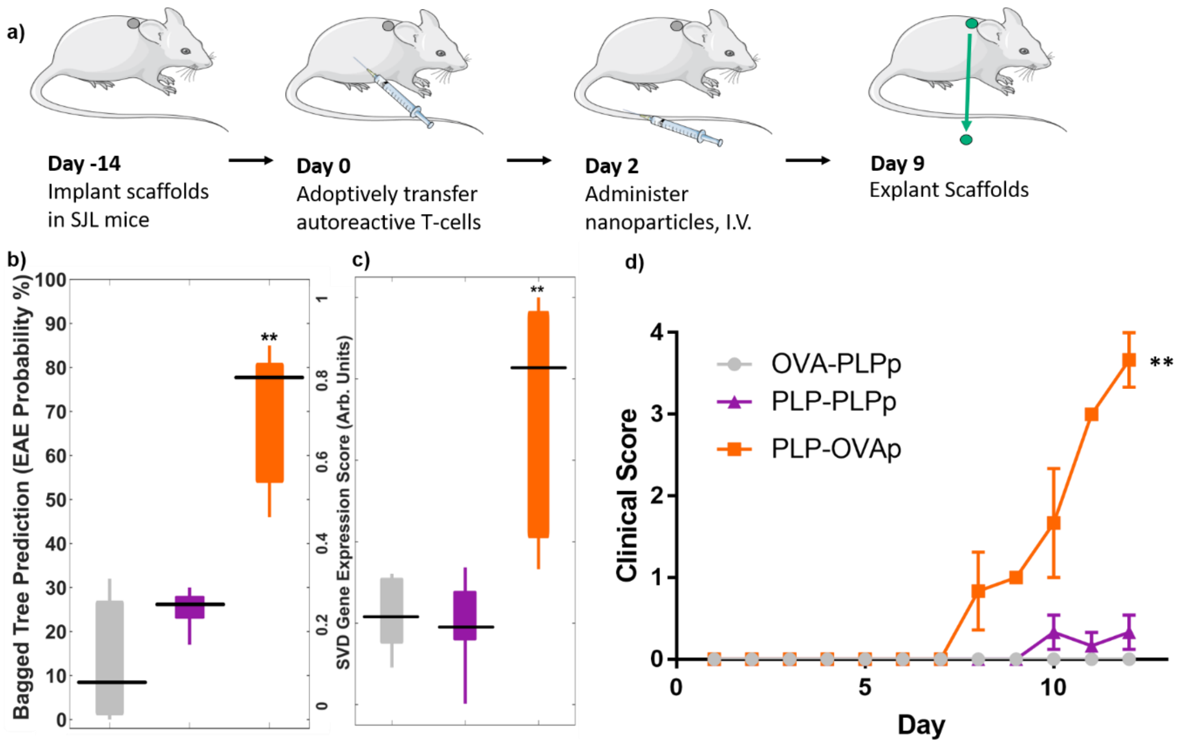

Supplementary Figure 13 IN enables treatment monitoring for nanoparticle induced tolerance. a) SJL mice were implanted with PCL INs 14 days prior to adoptive transfer of T cells reactive to either $PLP_{139-151}$ (EAE) or $OVA_{323-339}$ (control). Nanoparticle treatments were injected intravenously on day 2 and INs subsequently removed on day 9 for analysis via qPCR. Analysis of gene expression by the trained bagged tree ($F(2,15) = 34.2$, $p = 2.6e-06$) (b) and SVD ($F(2,15) = 15.96$, $p = 0.00020$) (c) demonstrated significantly higher scores for mice that received ineffective treatments (and developed symptoms of EAE). One-way ANOVA and a Bonferroni-corrected post-hoc multiple comparisons test was used to compare each group (**$p<0.0005$, different from both control and PLPp treated). d) Mean clinical scores of the mice at each time point demonstrate only the OVA loaded particle treatment demonstrating significantly increased scores from control. A Kruskal-Wallis test (one-way ANOVA on ranks) was used to compare scores for EAE mice after symptomatic onset (Kruskal-Wallis statistic = 11.69, $p=0.0002$) and a Dunn's multiple comparisons test was used to compare each group to the OVA control (**$p = 0.0016$). n=6 mice per group (three OVAp treated mice were euthanized at day 8). Box plots show the median, 25th-75th percentiles and most extreme data points not considered outliers (outliers are indicated by red +). Mouse and syringe cartoon from Servier Medical Art, https://smart.servier.com/smart_image/.

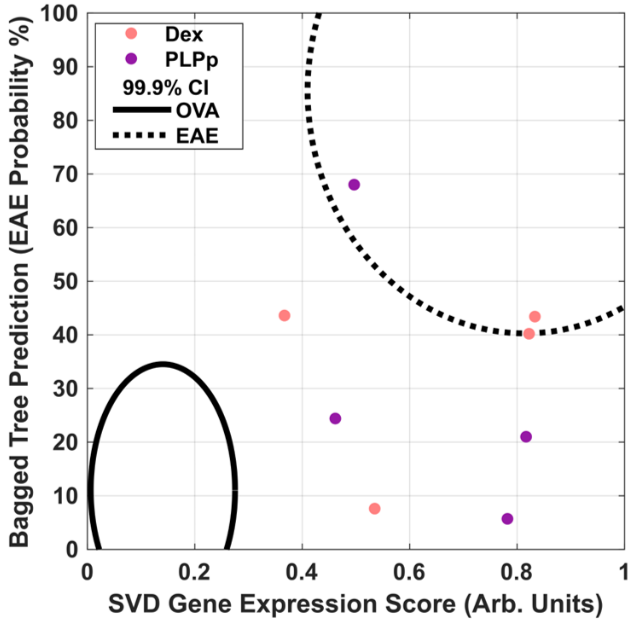

Supplementary Figure 14 INs indicate disease onset and enable pre-emptive treatment. INs were implanted subcutaneously two weeks before adoptive transfer to induce disease. Seven days post-transfer INs were explanted and gene expression analyzed to plot BT score versus SVD which indicated that mice were developing disease. This information was used to enable pre-emptive treatment with either an I.V. injection of 2.5 mg PLGA nanoparticles encapsulating $PLP_{139\text{-}151}$ or daily I.P. injections of 5 mg/kg dexamethasone.

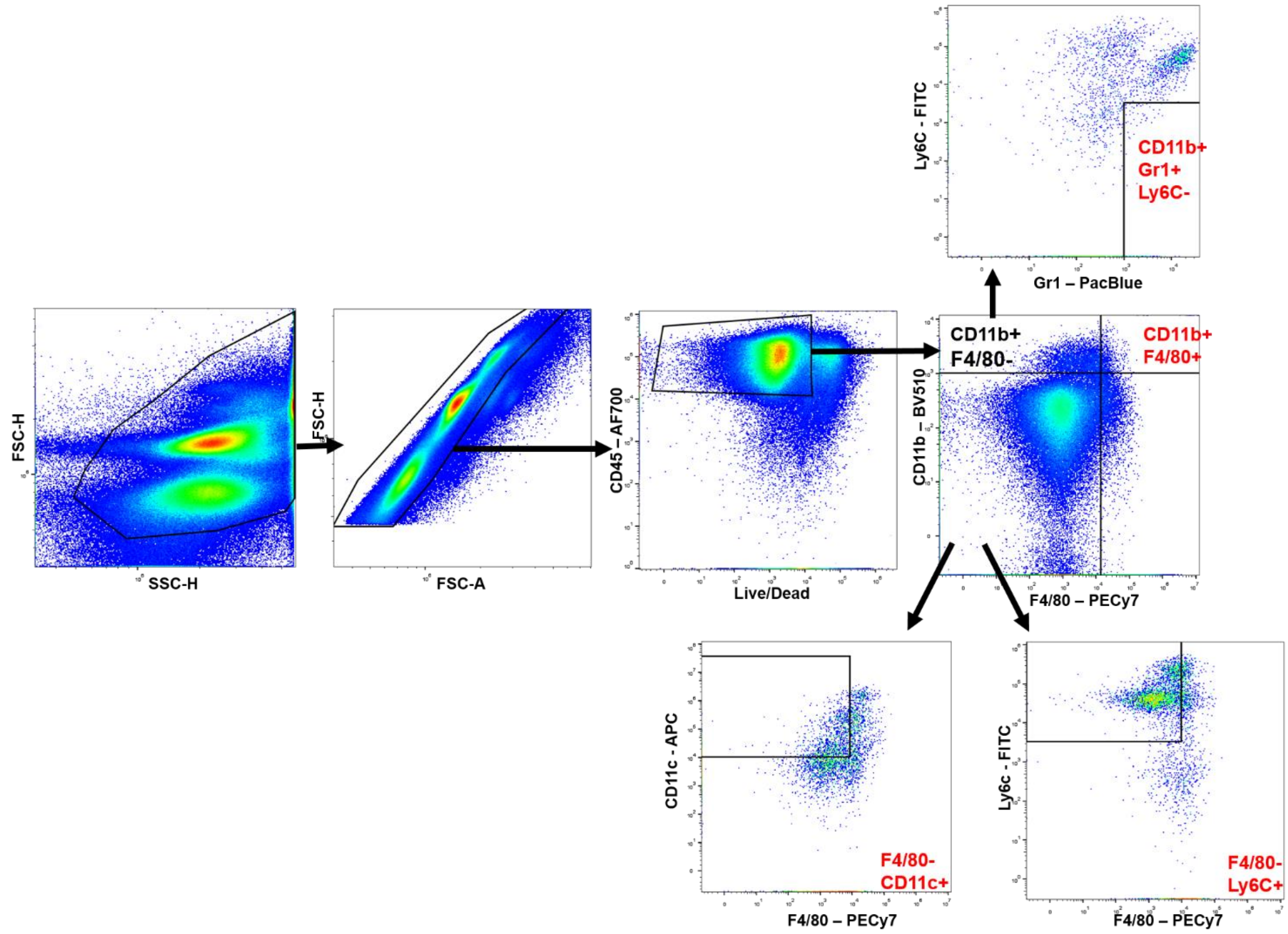

Supplementary Figure 15 Gating Scheme for innate immune panel. All samples were divided in half and half allocated to innate immune panel staining and half to adaptive. Cells were gated using forward scatter area and side scatter height before singlets were gated with forward scatter height and area. Within the singlet population CD45+, live cells (dead cell negative) stains were gated. At this point, CD11b and F480 were plotted to enable selection of CD11b+ F4/80+ cells and subsequent analysis. The CD11b+ gate was used to select Ly6C and Gr-1 to enable the selection of CD11b+ Gr-1+ Ly6C- cells. Then the F4/80 was plotted against Ly6C to enable selection of F4/80- Ly6C+ cells. Finally F4/80 was plotted with CD11c to enable selection of F4/80- CD11c+ cells. Arrows indicate flow of gating and red texts indicates final cell populations used for analysis.

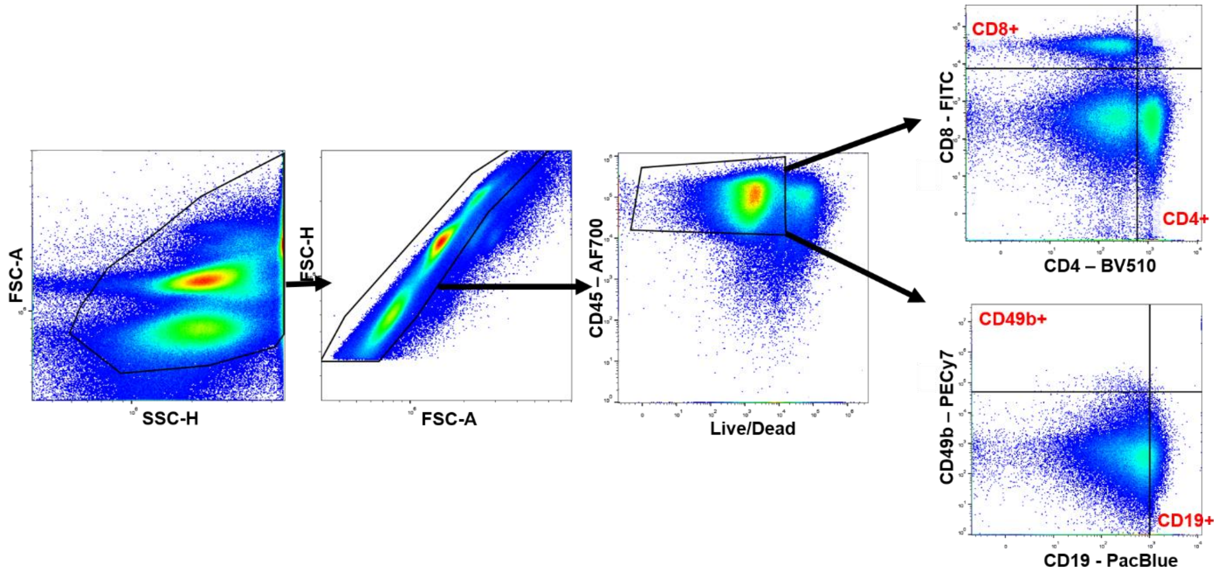

Supplementary Figure 16 Gating Scheme for adaptive immune panel. All samples were divided in half and half allocated to innate immune panel staining and half to adaptive. Cells were gated using forward scatter area and side scatter height before singlets were gated with forward scatter height and area. Within the singlet population CD45+, live cells (dead cell negative) stains were gated. At this point, CD4 and CD8 were plotted to enable selection of CD4+ or CD8+ cells. Next CD19 and CD49B were plotted to select the CD19+ and CD49b+ populations. Arrows indicate flow of gating and red texts indicates final cell populations used for analysis.

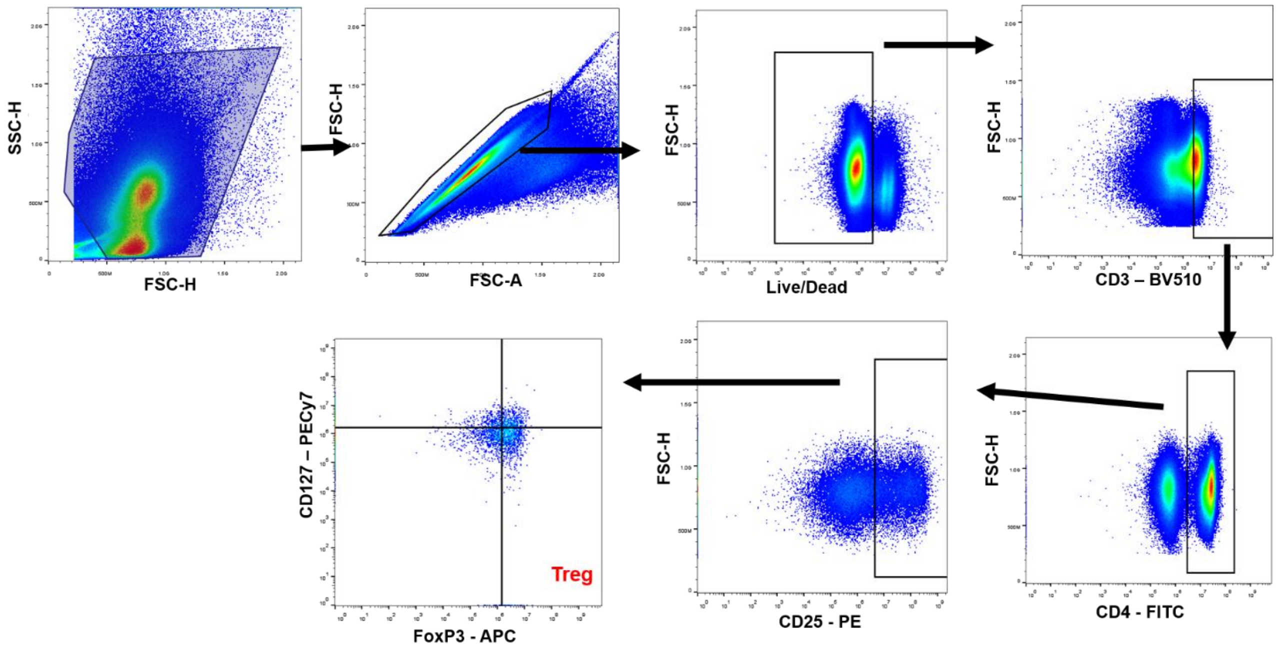

Supplementary Figure 17 Gating scheme for Treg panel. All samples were divided in half and half allocated to Treg panel staining and half to Th panel. Cells were gated using forward scatter and side scatter height before singlets were gated with forward scatter height and area. Within the singlet population live cells (dead cell negative) stains were gated. At this point, CD3 positive cells were selected of which the CD4 positive subset were selected for further analysis. CD25+ T-cells were selected. Finally, CD127 and FoxP3 were plotted to select the CD127- and FoxP3+ population as regulatory T-cells. Arrows indicate flow of gating and red texts indicates final cell populations used for analysis.

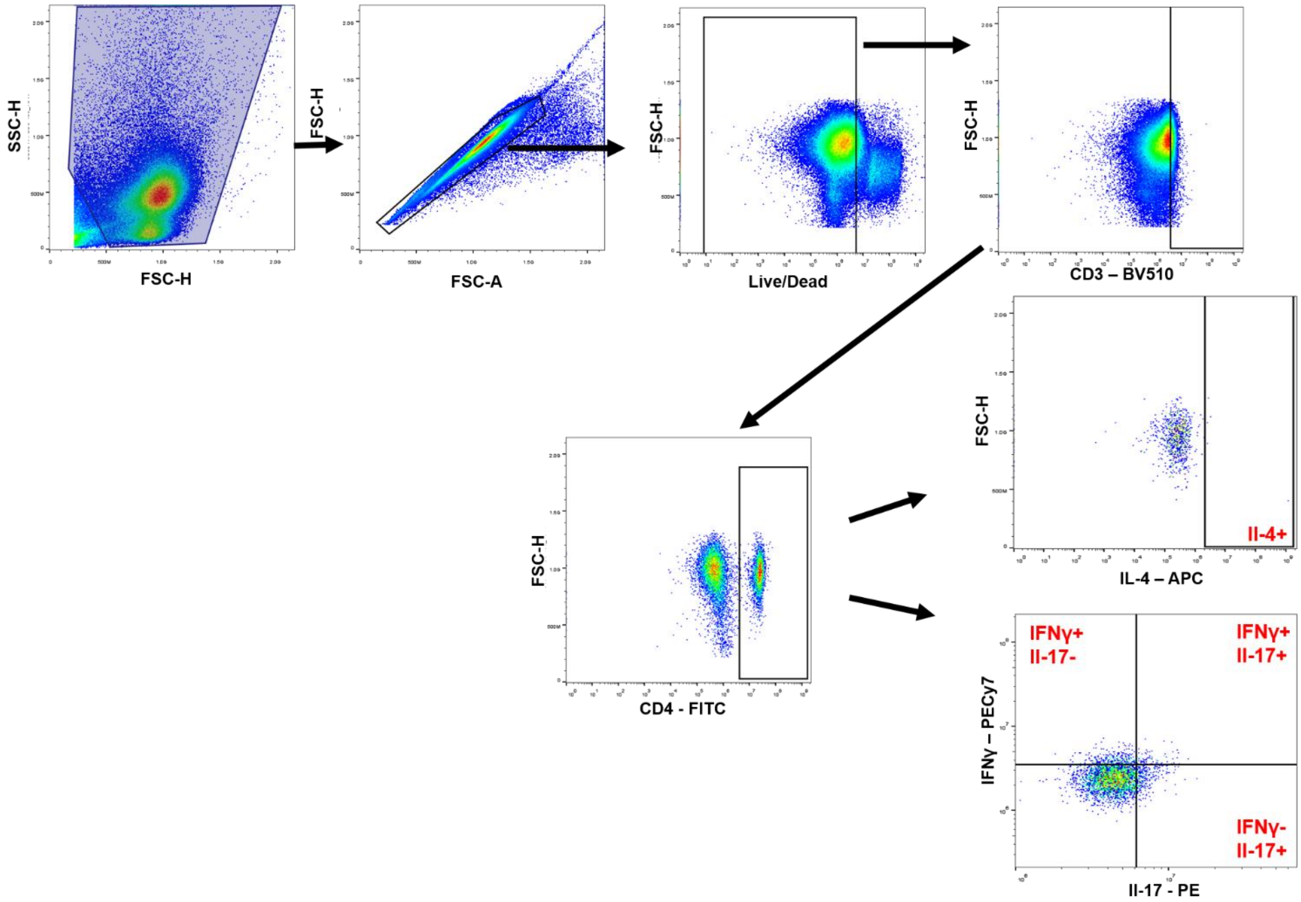

Supplementary Figure 18 Gating scheme for Th panel. All samples were divided in half and half allocated to Treg panel staining and half to Th panel. Cells were gated using forward scatter and side scatter height before singlets were gated with forward scatter height and area. Within the singlet population live cells (dead cell negative) stains were gated. At this point, CD3 positive cells were selected of which the CD4 positive subset were selected for further analysis. The CD4+ gate was used to select Il-4+ to enable the identification of Th2 cells. Finally, the CD4+ population was analyzed by plotting IFNγ stain against IL-17 to enable identification of other Th1/Th17 cell subsets. Arrows indicate flow of gating and red texts indicates final cell populations used for analysis.

Supplemental Tables:

Supplementary Table 1: p-values for Figure 3

	p-value (vs Untreated)	
Day	Dex	PLPp
1	1	1
2	1	1
3	1	1
4	1	1
5	1	1
6	1	1
7	1	1
8	0.13	0.28
9	0.0001	0.00028
10	0.0001	0.00047
11	0.0001	0.0001
12	0.0001	0.0001
13	0.0001	0.0001
14	0.0001	0.0001
15	0.0001	0.0001
16	0.0013	0.0001
17	0.002	0.0001
18	0.0013	0.0001

Supplementary Table 2: p-values for Supplementary Figure 2

	p-value PLP vs OVA		
	Pooled	d7	d9
Adrb2	0.0036	1.0	0.0021
Bdkrb1	0.0002	0.036	0.0086
Cd163	0.0029	0.082	0.2
Cfb	0.00014	0.6	0.00037
Clec7a	1.7E-05	0.071	0.00012
Cxcl1	0.0014	0.0027	1.0
Cxcl2	0.00072	0.045	0.049
Cxcl3	4.3E-05	0.015	0.0018
Cxcl5	3.3E-06	0.0085	0.000055
Ereg	0.0012	0.077	0.062
Fn1	0.00065	1.0	0.00066
Il1b	6.7E-05	0.024	0.0023
Il1f9	0.00014	0.045	0.0042
Il6	0.0044	0.041	0.69
Lif	0.011	0.14	0.71
Olr1	3.2E-06	0.015	0.000035
Ptgs2	1.3E-05	0.035	0.00014
S100a9	0.0014	0.055	0.11
Tnfrsf11b	1.3E-05	0.039	0.00013
Trem1	6.8E-05	0.014	0.004
Vegfa	0.2	1.0	0.33
