# Supplementary material for: Engineered immunological niches to monitor disease activity and treatment efficacy in relapsing multiple sclerosis
Source: Nat Commun. 2020 Aug 3;11:3871. doi: 10.1038/s41467-020-17629-z (PMC7398910; doi:10.1038/s41467-020-17629-z)
Supplement: Supplementary file 3 — Reporting Summary [file 41467_2020_17629_MOESM3_ESM.pdf]

nature research

Corresponding author(s): Lonnie Shea

Last updated by author(s): 7/1/2020

Reporting Summary

Nature Research wishes to improve the reproducibility of the work that we publish. This form provides structure for consistency and transparency in reporting. For further information on Nature Research policies, see Authors & Referees and the Editorial Policy Checklist.

Statistics

For all statistical analyses, confirm that the following items are present in the figure legend, table legend, main text, or Methods section.

n/a	Confirmed	
☐	☒	The exact sample size (n) for each experimental group/condition, given as a discrete number and unit of measurement
☐	☒	A statement on whether measurements were taken from distinct samples or whether the same sample was measured repeatedly
☐	☒	The statistical test(s) used AND whether they are one- or two-sided<br>*Only common tests should be described solely by name; describe more complex techniques in the Methods section.*
☒	☐	A description of all covariates tested
☐	☒	A description of any assumptions or corrections, such as tests of normality and adjustment for multiple comparisons
☐	☒	A full description of the statistical parameters including central tendency (e.g. means) or other basic estimates (e.g. regression coefficient) AND variation (e.g. standard deviation) or associated estimates of uncertainty (e.g. confidence intervals)
☐	☒	For null hypothesis testing, the test statistic (e.g. F, t, r) with confidence intervals, effect sizes, degrees of freedom and P value noted<br>*Give P values as exact values whenever suitable.*
☒	☐	For Bayesian analysis, information on the choice of priors and Markov chain Monte Carlo settings
☒	☐	For hierarchical and complex designs, identification of the appropriate level for tests and full reporting of outcomes
☒	☐	Estimates of effect sizes (e.g. Cohen's d, Pearson's r), indicating how they were calculated

Our web collection on statistics for biologists contains articles on many of the points above.

Software and code

Policy information about availability of computer code

Data collection	Applied Biosystems Quantstudio (v1.3) software was used to collect qPCR data.
Data analysis	Statistical analysis was performed in MATLAB (2017b) and Graphpad Prism (v7). Hierarchical clustering, SVD, and Bagged tree were performed in MATLAB using inbuilt MATLAB functions. FlowJo (v.X) was used in analysis of flow cytometry data.

For manuscripts utilizing custom algorithms or software that are central to the research but not yet described in published literature, software must be made available to editors/reviewers. We strongly encourage code deposition in a community repository (e.g. GitHub). See the Nature Research guidelines for submitting code & software for further information.

Data

Policy information about availability of data

All manuscripts must include a data availability statement. This statement should provide the following information, where applicable:
- Accession codes, unique identifiers, or web links for publicly available datasets
- A list of figures that have associated raw data
- A description of any restrictions on data availability

Data supporting the findings presented here are available within the paper and in the Supporting Information.

Field-specific reporting

Please select the one below that is the best fit for your research. If you are not sure, read the appropriate sections before making your selection.

☒ Life sciences ☐ Behavioural & social sciences ☐ Ecological, evolutionary & environmental sciences

Life sciences study design

All studies must disclose on these points even when the disclosure is negative.

Sample size	Sample sizes were not statistically predetermined, but were based on prior studies performed in the lab. Typically sample sizes of 4-6 have been sufficient for qPCR experiments (Oakes, et al. Cancer Res. 2019) and sample sizes of >6 have been appropriate for flow cytometry experiments (Rao, et al. Cancer Res. 2016; Aguado, et al. Acta Biomateriala. 2016). Each experimental group for in vivo studies contained at least these quantities of, but often more animals and were sufficient to obtain statistically significant differences between groups.
Data exclusions	Data were not excluded.
Replication	Each experiment/test was replicated at least 2-3 times. All attempts at replication were successful.
Randomization	All mice were syngeneic so randomization into groups was not necessary. For analysis of gene expression, samples were allocated into rows of plates randomly to account for any possible spatial variation in equipment.
Blinding	All studies were subject to quantitative analysis with the exception of determination of clinical scores (a somewhat subjective measurement). Clinical scores were always determined prior to any quantitative analysis of gene expression, etc. from samples to eliminate any bias in the scoring assessment. Additionally, A.H.M, K.R.H., and M.M.C. all scored mice throughout the study. Any borderline scores, were verified by more than one investigator. Clinical scores of treated mice were verified by multiple investigators to minimize bias.

Reporting for specific materials, systems and methods

We require information from authors about some types of materials, experimental systems and methods used in many studies. Here, indicate whether each material, system or method listed is relevant to your study. If you are not sure if a list item applies to your research, read the appropriate section before selecting a response.

Materials & experimental systems

n/a	Involved in the study	
☐	☒	Antibodies
☒	☐	Eukaryotic cell lines
☒	☐	Palaeontology
☐	☒	Animals and other organisms
☒	☐	Human research participants
☒	☐	Clinical data

Methods

n/a	Involved in the study	
☒	☐	ChIP-seq
☐	☒	Flow cytometry
☒	☐	MRI-based neuroimaging

Antibodies

Antibodies used

anti-CD16/32 (clone 93, eBioscience, Catalog # 14-0161-82)
Alexa Fluor® 700 anti-CD45 (clone 30-F11, Biolegend, Catalog # 103128)
FITC anti-CD8 (clone 53-6.7, Biolegend, Catalog # 100705)
Pacific Blue™ anti-CD19 (clone 6D5, Biolegend, Catalog #115526)
FITC anti-Ly6C (clone HK1.4, Biolegend, Catalog # 128005)
Pacific Blue™ anti- Ly-6G/Ly-6C (Gr-1) (clone RB6-8C5, Biolegend, Catalog # 108429)
PE-Cy7 anti-F4/80 (clone BM8, Biolegend, Catalog # 123113)
V500 anti-CD11b (clone M1/70, BD Biosciences, Catalog # 562127)

V500 anti-CD4 (clone RM4-5, BD Biosciences Catalog # 560782)
PE-Cy7 anti-CD49b (clone DX5, Biolegend, Catalog # 108921)
APC anti-CD11c (clone N418, Biolegend, Catalog # 117309)

BV510 anti-CD3 (clone 17A2, Biolegend, Catalog # 100233)
FITC anti-CD4 (clone RM4-5, Biolegend, Catalog # 100509)
PE anti-CD25 (clone PC61, Biolegend, Catalog # 102007)
APC anti-FoxP3 (clone FJK-16s, eBioscience, Catalog # 17-5773-82)
PE-Cy7 anti-CD127 (clone A7R34, Biolegend, Catalog # 135013)
PE anti-IL-17a (clone TC11-18H10.1, Biolegend, Catalog # 506903)
APC anti-IL-4 (clone 11B11, Biolegend, Catalog # 504105)
PE-Cy7 anti-IFNγ (clone XMG1.2, Biolegend, Catalog # 505825)

Validation

All antibodies used were commercially validated.
All Biolegend antibodies: "Each lot of this antibody is quality control tested by immunofluorescent staining with flow cytometric analysis." These antibodies include:

Alexa Fluor® 700 anti-CD45 (clone 30-F11, Biolegend, Catalog # 103128)
FITC anti-CD8 (clone 53-6.7, Biolegend, Catalog # 100705)
Pacific Blue™ anti-CD19 (clone 6D5, Biolegend, Catalog #115526)
FITC anti-Ly6C (clone HK1.4, Biolegend, Catalog # 128005)
Pacific Blue™ anti- Ly-6G/Ly-6C (Gr-1) (clone RB6-8C5, Biolegend, Catalog # 108429)
PE-Cy7 anti-F4/80 (clone BM8, Biolegend, Catalog # 123113)
PE-Cy7 anti-CD49b (clone DX5, Biolegend, Catalog # 108921)
APC anti-CD11c (clone N418, Biolegend, Catalog # 117309)
FITC anti-CD4 (clone RM4-5, Biolegend, Catalog # 100509)
PE anti-CD25 (clone PC61, Biolegend, Catalog # 102007)
PE-Cy7 anti-CD127 (clone A7R34, Biolegend, Catalog # 135013)
PE anti-IL-17a (clone TC11-18H10.1, Biolegend, Catalog # 506903)
APC anti-IL-4 (clone 11B11, Biolegend, Catalog # 504105)
PE-Cy7 anti-IFNγ (clone XMG1.2, Biolegend, Catalog # 505825)

eBioscience antibodies validated against isotype control. These antibodies include:
APC anti-FoxP3 (clone FJK-16s, eBioscience, Catalog # 17-5773-82)
anti-CD16/32 (clone 93, eBioscience, Catalog # 14-0161-82)

BD Biosciences antibody, "The production process underwent stringent testing and validation to assure that it generates a high-quality conjugate with consistent performance and specific binding activity". These antibodies include:
V500 anti-CD11b (clone M1/70, BD Biosciences, Catalog # 562127)
V500 anti-CD4 (clone RM4-5, BD Biosciences Catalog # 560782)

Animals and other organisms

Policy information about studies involving animals; ARRIVE guidelines recommended for reporting animal research

Laboratory animals	Female SJL/J mice at six weeks
Wild animals	No wild animals were used in this study.
Field-collected samples	No field-collected samples were used in this study.
Ethics oversight	All animal procedures were performed in accordance with the regulations approved by and guidance of the Animal Care and Use Committee of the University of Michigan.

Note that full information on the approval of the study protocol must also be provided in the manuscript.

Flow Cytometry

Plots

Confirm that:

☒ The axis labels state the marker and fluorochrome used (e.g. CD4-FITC).

☒ The axis scales are clearly visible. Include numbers along axes only for bottom left plot of group (a 'group' is an analysis of identical markers).

☒ All plots are contour plots with outliers or pseudocolor plots.

☒ A numerical value for number of cells or percentage (with statistics) is provided.

Methodology

Sample preparation	Briefly, samples were minced with a scalpel and incubated for 20 minutes in Liberase TL (Roche) at 37 °C. TSs were then mashed through a 70 µm filter which was washed extensively with FACS buffer: PBS (Life Technologies) with 0.5% Bovine Serum Albumin (Sigma Aldrich) and 2 mM EDTA (Gibco). Cells were equally split into two tubes to enable staining and analysis of innate and adaptive immune cells from the same TS and then blocked with anti-CD16/32 (1:50, clone 93, eBioscience). Each tube was stained with Live/Dead Fixable Red (Life Technologies) and Alexa Fluor® 700 anti-CD45 (1:125, clone 30-F11, Biolegend). The adaptive immune panel was also stained with: FITC anti-CD8 (1:25, clone 53-6.7, Biolegend), Pacific Blue™ anti-CD19 (1:100, clone 6D5, Biolegend), PE-Cy7 anti-CD49b (1:30, clone DX5, Biolegend), and V500 anti-CD4 (1:100, clone RM4-5, BD Biosciences). The innate immune panel was also stained with: APC anti-CD11c (1:80, clone N418, Biolegend), FITC anti-Ly6C (1:100, clone HK.14, Biolegend), Pacific Blue™ anti- Ly-6G/Ly-6C (Gr-1) (1:70, clone RB6-8C5, Biolegend), PE-Cy7 anti-F4/80 (1:80, clone BM8, Biolegend), and V500 anti-CD11b (1:100, clone M1/70, BD Biosciences). Samples were analyzed on a Cytoflex Cell Analyzer, and all single color controls and FMOs were used to aid with gating and compensation.
Instrument	Cytoflex Cell Analyzer
Software	FlowJo (v. X)
Cell population abundance	No sorting occurred (only flow cytometry). Therefore, there is no data regarding "post-sort fractions"

Gating strategy

Populations were gated based on FMOs

☒ Tick this box to confirm that a figure exemplifying the gating strategy is provided in the Supplementary Information.
